# Endovascular Management of Hemorrhagic Stroke

**DOI:** 10.3390/biomedicines10010100

**Published:** 2022-01-04

**Authors:** Basel Musmar, Nimer Adeeb, Junaid Ansari, Pankaj Sharma, Hugo H. Cuellar

**Affiliations:** Department of Neurosurgery and Neurointerventional Radiology, Louisiana State University, Shreveport, LA 70803, USA; naa001@lsuhs.edu (B.M.); nimer@uab.edu (N.A.); junaid.ansari@lsuhs.edu (J.A.); pankaj.sharma@lsuhs.edu (P.S.)

**Keywords:** stroke, hemorrhage, SAH, aneurysms, arteriovenous, malformation, fistula

## Abstract

Significant advances in endovascular neurosurgery tools, devices, and techniques are changing the approach to the management of acute hemorrhagic stroke. The endovascular treatment of intracranial aneurysms emerged in the early 1990s with Guglielmi detachable coils, and since then, it gained rapid popularity that surpassed open surgery. Stent-assisted coiling and balloon remodeling techniques have made the treatment of wide-necked aneurysms more durable. With the introduction of flow diverters and flow disrupters, many aneurysms with complex geometrics can now be reliably managed. Arteriovenous malformations and fistulae can also benefit from endovascular therapy by embolization using n-butyl cyanoacrylate (NBCA), Onyx, polyvinyl alcohol (PVA), and coils. In this article, we describe the role of endovascular treatment for the most common causes of intracerebral and subarachnoid hemorrhages, particularly ruptured aneurysms and vascular malformations.

## 1. Introduction

The impact of stroke on Americans is enormous. Stroke is the fifth leading cause of death and a leading cause of disability in the United States, with an incidence of approximately 800,000 new strokes every year, which is approximately one new stroke every 40 s [1]. 

Stroke can be classified into two types. The first type is ischemic stroke, which is more common and accounts for 85% of all acute strokes; ischemic stroke is caused by the interruption of blood flow to a certain area of the brain. The second type is hemorrhagic stroke, which accounts for 15% of acute strokes and is caused by the rupture of a blood vessel. Hemorrhagic stroke can also be divided into two main types: intracerebral hemorrhage (ICH) and subarachnoid hemorrhage (SAH).

ICH is diagnosed more frequently in the elderly (>55 years of age), and it is more common in men than in women with a predilection in the African and Asian populations [2,3,4]. Although the mortality rate related to ICH has decreased worldwide [5], its incidence in low/middle-income regions is doubled (50 per 100,000) compared to the rates in more economically developed countries [2]. The most important risk factors of ICH are increased age and chronic hypertension [3,4]. Other etiologies include amyloid angiopathy, anticoagulation (medication), vascular malformations, ruptured aneurysms, coagulopathies, hemorrhagic transformation of an arterial or venous infarct, malignancy, drug abuse, and vasculitis [3,4]. Treatment of ICH is divided into medical management and surgical evacuation. Endovascular management is limited to vascular malformation etiologies, which will be discussed later. 

SAH affects approximately 30,000 individuals annually in the Unites States and accounts for about five percent of all strokes [6,7,8]. The incidence of SAH in the United States is between 10 to 14 for 100,000 population per year [9]. It is the only stroke type more common in women and it is more prevalent in black and Hispanic populations than in white populations [9]. 

The most common etiologies for SAH are trauma and intracranial aneurysms [4,9]. Trauma causes the highest incidence of SAH [9]. Aneurysms and other non-traumatic causes including arteriovenous malformations (AVMs) and arteriovenous fistulas (AVFs), vasculopathy and arterial dissection should be suspected when the patient presents with spontaneous SAH [4,9]. It is noteworthy that approximately 15–30% of SAH are idiopathic despite complete evaluation [10]. 

Endovascular treatment has gradually become a first-line treatment of intracranial aneurysms and other vascular malformations since the first detachable coils, the Guglielmi detachable coils (GDC) [11,12,13]. Here in, we will describe the endovascular management for the most common causes of ICH and SAH.

## 2. Ruptured Intracranial Aneurysms 

### 2.1. Background

The estimated prevalence of intracranial aneurysms in the general population ranges from 0.4% to 3% [14]. Most aneurysms are asymptomatic and are incidentally found on angiographic imaging [15]. Other common presentations of intracranial aneurysms include headaches, mass effect on a cranial nerve, or embolic stroke due to thrombus formation within the aneurysm sac. Still, the most feared presentation is aneurysm rupture, leading to SAH or ICH formation. 

Most aneurysms are saccular in shape and account for nearly 85% of nontraumatic SAHs [16]. Other types include fusiform, mycotic, and dissecting. The risk of aneurysm rupture is multifactorial, but patients who smoke, have hypertension, and have a family history of SAH or other predisposing condition, such as polycystic kidney disease or connective tissue disease, have a higher risk for rupture. Those patients also tend to have aneurysm size greater than 7 mm [17]. 

### 2.2. Management

The first goal of endovascular treatment of ruptured aneurysms is occlusion of the aneurysm and prevention of rebleeding. Endovascular treatment has gradually overtaken surgical clipping as the mainstay treatment of ruptured aneurysms in the light of the results of the International Subarachnoid Aneurysm Trial (ISAT), the largest randomized, prospective trial comparing endovascular techniques with open surgery in ruptured aneurysms [18]. This trial showed significantly better outcomes at 1 year for patients treated by endovascular therapy compared to open surgery, with nearly 23% relative risk reduction and 7% absolute risk reduction of death or disability measured using the modified Rankin Scale.

Indications and disadvantages of different treatment modalities are summarized in Table 1. 

### 2.3. Coiling

Endovascular therapy emerged in the early 1990s with the GDC [11,12]. These very flexible spiral coils are made of a platinum alloy, which is compatible with MRI. Since then, several technological developments aimed to develop coils with a wide variety of sizes, with the smallest coils of 1 mm and largest coils over 20 mm [19], 3D shapes, and flexibility that can adapt all types of aneurysms. A large multicenter series evaluated the feasibility of GDC coils on over 700 ruptured aneurysms [20]. Coiling was feasible in 96.9% of cases with a mortality rate of 1.4% and morbidity of 8.6%. 

The most common and preferred technique to treat aneurysms through an endovascular approach is the constructive approach [4]. The aneurysm is fully occluded with coils while flow through parent artery is preserved. Sometimes constructive endovascular or surgical approaches are not safe or feasible as in cases of dissecting, giant, or distal aneurysms, therefore, a deconstructive approach may be considered, in which the aneurysm is obliterated along with its parent artery. This can be done by packing an aneurysm and its parent artery with coils.

The most frequent complications of aneurysm coiling are thromboembolic complications and intraoperative rupture [21]. In ruptured aneurysms, the rates of thromboembolic complications and intraoperative rupture were 13.3% and 3.7%, respectively [22]. 

One of the disadvantages of aneurysm coil embolization is the relatively high recanalization rate. A systematic review of a large number of studies for coil embolization of intracranial aneurysms showed that aneurysm recanalization occurred in 20.8% of cases, requiring retreatment in 10.3% [23]. The risk of recanalization is higher in bifurcation aneurysms, mainly basilar tip aneurysms, reaching up to 33% [24,25]. Several factors were identified to be associated with an increased risk for recanalization, including recent rupture, high blood pressure, smoking, aneurysm diameter and neck size, and coil packing density [21]. 

Another challenge of coil embolization are aneurysms that are not easy to treat due to their shape, including large and giant aneurysms, fusiform aneurysms, and large neck aneurysms. 

### 2.4. Balloon-Assisted Coiling

Several techniques were adopted later to mitigate the limitations of coiling in treating wide neck aneurysms and their high risk of recanalization. The first technique was balloon remodeling technique, which primarily offers temporary balloon inflation during coil delivery to achieve homogenous packing density of wide-necked, complex-shaped cerebral aneurysms [26]. Moret et al. conducted a study to evaluate the safety of remodeling technique [26]. A total of 56 aneurysms in 54 patients were included, and 37 (70%) of the patients presented with SAH. The remodeling technique was reported to be used in 52 aneurysms successfully with complete occlusion rate of 77%. The patient mortality and procedure-associated morbidity were 0.5%. 

Cekirge et al. published a series of 800 patients with 864 aneurysms treated with HyperForm balloon assistance [27]. There were 647 patients (80.8%) with existing SAH. An initial Raymond-Roy 1 occlusion rate was achieved in 73% of aneurysms. The overall mortality rate was 7.1%, including 1.4% procedural mortality. The follow-up angiograms, which were obtained for 87.6% of patients showed a recanalization rate of 12%, further thrombosis in 17%, and class 1 occlusion in 82%. The retreatment rate was 9%.

Shapiro et al. conducted a literature review and meta-analysis to assess the adverse events of balloon assistance compared to coiling alone. They found no higher incidence of thromboembolic events or iatrogenic rupture with the use of balloon remodeling when compared to coiling alone [28]. 

### 2.5. Stent-Assisted Coiling 

Another technique to improve occlusion rate after coiling, prevent coil herniation, and reduce retreatment rate is stenting. Stents were developed to buttress the coil mass at the aneurysm neck while providing some degree of flow diversion, facilitating aneurysm thrombosis and occlusion [11]. Stents are particularly useful in large and wide-necked aneurysms not suitable for primary coiling alone. They result in complete exclusion of the aneurysm from the circulation by providing a scaffold for endothelial coverage of the aneurysm neck while preserving side branches and perforators [29,30]. 

In a large retrospective study by Piotin et al., permanent neurological procedure-related complications occurred in 7.4% of the procedures with stents versus 3.8% in the procedures without stents. Procedure-induced mortality occurred in 4.6% of the procedures with stents versus 1.2% in the procedures without stents. Angiographic recurrence occurred in 14.9% stent-assisted coiling versus 33.5% of coiled only aneurysms [31]. In another study by Jahshan et al. the authors observed similar permanent morbidity in the none-stenting arm as compared with the stenting group. However, a higher rate of complete occlusion was seen in the stented group [32]. 

### 2.6. Flow Diversion

More recently, stents with high surface-area coverage were introduced for intracranial aneurysm treatments, called flow diverters. Those flow diverters bridge the aneurysm neck and reduce the blood flow into the aneurysm sac yet provide blood flow through adjacent perforators and side branches. This creates a redirection of the blood flow away from the aneurysm. Reduction of blood circulation within the aneurysm leads to flow stasis and promotes the formation of a stable aneurysmal thrombus. Moreover, they provide a scaffold for neoendothelization across the aneurysm neck, and thus result in aneurysm exclusion from the circulation [21]. 

The Pipeline for Uncoilable or Failed Aneurysms (PUFS) trial was the first prospective, international multicenter series focused on treatment of complex aneurysms using the Pipeline Embolization Device (PED) [33]. The study concluded PED offers a safe and effective treatment of large or giant intracranial internal carotid artery aneurysms, demonstrated by high rates of complete aneurysm occlusion and low rates of adverse neurologic events. Later on, many studies have demonstrated the safety and efficacy of PED in treating aneurysms with varying morphologies and which are located in different anatomical locations [29,33,34,35,36,37,38,39], including posterior circulation aneurysms [40,41]. In a study by Adeeb et al. that included 465 aneurysms treated with PED, complete occlusion (100%) was achieved in 78.2% of aneurysms, while near complete occlusion (90–99%) was achieved in 7.6%, and partial occlusion (<90%) in 14.2%. Given that aneurysm occlusion rate continues to increase with time following flow diversion, the rate increased to 83.9% in aneurysms that were followed >12 months. The retreatment rate was 6.3%, with no incidence of recanalization after complete occlusion [42]. 

The unique mechanisms of action of the flow diverters require a high surface coverage ratio. This metallic coverage increases the potential for platelet activation and thrombus formation, making thromboembolic complications the most frequent cause of peri-procedural morbidity, reported in 4–9% of PED cases [21,43,44,45,46,47,48]. To minimize this risk, dual anti-platelet therapy is implemented pre-procedure and continued for at least 3 months post-procedure. 

Although flow diversion showed a favorable outcome for the treatment of unruptured aneurysms, the need for dual antiplatelet therapy represents a limitation of flow diverters in ruptured aneurysms. Cagnazzo et al. conducted a meta-analysis that included 20 studies evaluating 223 patients with acutely ruptured anterior or posterior circulation aneurysms treated with flow-diverter stents [49]. The rate of treatment-related complications in the study population was 17.8%, with a higher complication rate in posterior circulation aneurysms and in aneurysms treated with multiple stents compared to those treated with a single stent. In addition, aneurysm rebleeding in this treated cohort was 4.0% and was highest in the first 72 h post-procedure. [49] On several occasions, the use of flow diverters is selected in certain indications for ruptured aneurysms such as dissecting aneurysms and “blister like” aneurysms [50,51]. 

### 2.7. Flow Disruption

Recently, a new concept of intrasaccular flow disruption was developed. The approach is similar to the intraluminal flow diversion technology; however, the mesh of the flow disruptor is placed within the aneurysm pouch and creates blood flow stasis with subsequent thrombosis. The first and most commonly used intrasaccular device is the Woven EndoBridge (WEB) system (Sequent Medical Inc, Aliso Viejo, CA, USA). Recent studies showed up to 84.6% of adequate occlusion rate, with low morbidity and mortality [52,53,54,55]. The WEB device seems to be well suited for the treatment of wide-neck bifurcation aneurysms of the basilar artery, the middle cerebral artery, the anterior communicating artery, and the internal carotid artery. 

In contrast to the use of stents and neck bridging devices, dual antiplatelet medication is not necessary for the aneurysm treatment with the WEB, as the device is designed to stay within the limits of the aneurysm [56]. This makes the use of the WEB device more ideal for ruptured aneurysms that are difficult to treat by standard endovascular or open surgical approaches. 

Recently, an international multicenter collaboration known as WorldWideWEB consortium is ongoing to assess the safety and efficacy for the use of WEB device for treatment of intracranial aneurysms in large real-world data.

### 2.8. Things to Consider

*Screening:* The most preventable risk factors for aneurysm formation are smoking and untreated hypertension, which may raise the risk up to four- and eightfold, respectively [57]. The most important unpreventable risk factor is family history. First-degree relatives have a lifetime risk of an aneurysm of approximately 10%, with up to a sevenfold greater risk of SAH [58], which may be affected by age, gender, racial or ethnic background, and the presence of a predisposing condition. It is noteworthy that familial aneurysms tend to be larger and more likely to occur in multiple locations [59]. For aneurysms with size 3 mm or larger, magnetic resonance angiography (MRA) or CT angiography are generally adequate for evaluation [4]. Physicians should keep in mind that a negative result in screening does not preclude aneurysm formation in the future. 

*Timing of treatment:* Treatment of ruptured aneurysms within 24 h is associated with improved clinical outcomes compared with treatment at >24 h [60]. The benefit is more pronounced for coiling than clipping.

## 3. Arteriovenous Malformations 

### 3.1. Background

As the name implies, AVMs are vascular malformations composed of a network of abnormal vessels connecting directly between an artery and a vein without intervening capillary bed. These malformations are usually congenital lesions originated from persistent primitive arteriovenous connections [61]. 

The incidence of cerebral arteriovenous malformations ranges between 0.8 and 1.3 per 100,000 person years [62,63,64]. Arteriovenous malformations can be associated with syndromes like hereditary hemorrhagic telangiectasia, Sturge Weber syndrome, and Wyburn-Mason syndrome [65,66]. AVMs have various clinical presentations: the most common are hemorrhage (50%), seizures (33%), headache (16%), or focal neurologic deficit (6%) [67].

When ruptured, AVMs most often cause intraparenchymal hemorrhage; however, subarachnoid and subdural hemorrhages may still occur [4]. Annual mortality rate is approximately 1.5% and survivors have a 10–30% long-term disability [68]. The risk of hemorrhage is associated with many risk factors like high intranidal pressure, deep location, exclusive deep venous drainage and single draining vein, the presence of associated aneurysms or pseudoaneurysms, and prior history of intracranial bleeding due to AVM [61,67,69]. A recent hemorrhage may raise the risk of rebleeding in the first year to approximately 32% and decreases to 11% in subsequent years [70]. Due to the higher likelihood of a lobar hematoma when compared to ruptured aneurysms, the neurological disability is more common in ruptured AVMs [68]. Spetzler et al. studied the relationship between the size of an arteriovenous malformation and its propensity to hemorrhage. They found that smaller AVMs (<3 cm) have significantly higher feeding artery pressures than larger AVMs (>6 cm) and are, thus, associated with large hemorrhages [71]. 

### 3.2. Management 

The main goal of the management of AVM is to obliterate it and diminish the risk of future neurologic events, especially hemorrhage [72]. There were significant advances in endovascular management over the past years, from the first embolization of an AVM by Luessenhop and Spence et al. in 1960 [73], and selective catheterization of the intracranial circulation with microcatheters by Serbinenko et al. [74] to embolization agents like N-butyl cyanoacrylate (NBCA) and ethylene vinyl alcohol polymer (Onyx, Medtronic, Irvine, CA, USA). All those advancements have allowed increasingly complex AVMs to be treated. Endovascular treatment therapy can also be used before open surgery to treat large AVMs to reduce intraoperative blood loss, decrease surgery time, decrease the size of an AVM, the Spetzler-Martin grade, and eventually decreased morbidity-mortality [61,75]. On the other hand, small AVMs can be completely cured by endovascular therapy alone [76]. Complete obliteration rates with embolization have been reported between 9.7 and 14% with NBCA [77]. With the introduction of the new agent, Onyx, complete obliteration rates rose to 18–51% [78]. As well, endovascular therapy can be used prior to radiosurgery in order to shrink the nidus, thus, decreasing the risk of hemorrhage until the lesion is completely obliterated. Some studies reported a decrease in obliteration rate if pre-radiation embolization was performed [79,80], while others have demonstrated increased efficacy of radiosurgery with better obliteration rates when used after embolization [81]. 

Sometimes aneurysms and pseudoaneurysms can arise from the nidus, feeding arteries, and along the draining veins of an AVM. This can increase the flow rate, making it more turbulent, therefore, increasing the chance of hemorrhage [82]. They may be treated by embolization or clipping if they are distant from the nidus [4]. 

Prior to endovascular treatment, operators need to define the expected goal of the procedure, whether it is complete obliteration, size reduction before radiosurgery, presurgical grade reduction, or to obliterate weak angioarchitectural points (i.e., aneurysms/pseudoaneurysms). For instance, if the goal is a cure or significant size reduction, the ideal position of the microcatheter will be as distal as possible and close to the nidus of the AVM, and so forth [61]. After setting the main goal of treatment, a plan can be organized. 

In general, the main target of embolization is the AVM nidus. Occlusion of either arterial feeders or venous outflow will make the situation worse. If the arterial feeders were occluded alone, this will lead to a temporary decrease in shunting and delayed recruitment of new arterial supply while occlusion of venous outflow will lead to immediate disastrous hemorrhage from continued arterial inflow and uncontrolled hypertension [4]. Even though a venous outflow occlusion is one of the most feared situations during AVM embolization, there is an increasing trend toward using transvenous embolization in selective cases especially when the AVM is deeply located with small tortious feeders or a complex anatomy of the passage feeders, making the use of transarterial embolization difficult [83,84,85]. Embolization using the transvenous approach has many “theoretical” advantages, including better penetration of the AVM nidus, less ischemic events, and relatively easier navigation through enlarged and usually straighter veins [61]. Studies showed a complete obliteration between 80% and 100% [85,86]. Theoretical criteria to choose the patients who can benefit the most from this approach include small AVM with a nidus of <2 cm, hemorrhagic AVMs, arterial feeders that are not amenable to transarterial embolization, lenticulostriate arteries or choroidal arteries, AVMs with a single drainage vein, and patients who are good surgical candidates [84,86]. 

The decision between embolization agents is driven by the angioarchitectural characteristics and preferences and experience of the practitioner. The most commonly used embolization agents are Onyx and NBCA. Onyx is superior to NBCA in injection times (longer) and possibility of halting injections with intermittent angiographic control, though, NBCA is still preferred by some institutions [61,87]. Onyx is equivalent to NBCA in safety, efficacy, and in the capacity for at least 50% volume reduction [88]. 

NBCA is an adhesive agent that polymerizes to a solid form when it comes into contact with blood. It is typically mixed with Ethiodol in concentrations of 1:3 or 1:2 (NBCA:Ethiodol), depending on the desired viscosity. Denser mixtures can be used in high flow rates while diluted mixtures can penetrate better into the nidus with the throwback of higher chance of reflux [61]. 

In addition to Onyx and NBCA, other FDA approved materials are available to achieve embolization, including polyvinyl alcohol (PVA) and platinum coils. PVA is mixed into slurry with contrast, occludes vessels and causes an inflammatory reaction and angiogenesis [89]. It is more often used in conjugation with coils. The popularity of PVA is declining due to the success of NBCA and high recurrence rate after PVA occlusion. A study that evaluated PVA in terms of recanalization and complication rate found out that recanalization is a distinct possibility with a percentage of up to 43% at 1 month and up to 80% overall even when there was an initial cure. [90] Other non-FDA approved materials are Squid (Emboflu, Gland, Switzerland) and Precipitating Hydrophobic Injectable Liquid (PHIL; MicroVention, Tustin, CA, USA). Squid is an ethylene vinyl alcohol copolymer (EVOH) that has become available recently. It has two formulations: Squid 12 with less viscous formulation for improved vascular penetration and Squid LD with a lower density formulation compared to Onyx [91]. Akmangit et al. evaluated 28 patients who were treated with Squid [91]. The total obliteration rate of the AVMs was 37.5%. There was no mortality. Two reported hemorrhages and thromboembolic complications resulted in permanent deficits in three patients.

PHIL is a new non-adhesive liquid embolic agent comprising a copolymer dissolved in dimethylsulfoxide (DMSO). The PHIL liquid embolic system is available in three formulations: 25%, 30%, and 35%. [92] A retrospective multicenter study assessed the efficacy and safety of PHIL [92]. Twenty-two (85%) patients were treated with PHIL only, with three patients treated with both PHIL and Onyx, and one with both PHIL and coils. Immediate complete angiographic occlusion was achieved in 20 (77%) patients. An adverse event was seen in one patient who developed a worsening of pre-existing ataxia due to acute thrombosis of the draining vein. 

### 3.3. Complications

The overall morbidity and mortality of AVM embolization range from 0% to 22% and from 0% to 3%, respectively [93,94,95,96,97]. The most serious complications are hemorrhagic and ischemic stroke. Intracranial hemorrhage during or after embolization can be seen in between 2% and 4.7% [61]. The most common cause of hemorrhage is vessel perforation from a microwire used to guide the microcatheter [61]. In addition, hemorrhage may occur as a result of the rupture of the AVM nidus or a pre- or intranidal aneurysm when a major draining vein is occluded [4]. Ischemic complications can occur due to thromboembolism from the embolic material or thrombus formation within or along the guiding catheter or microcatheter [61]. 

In addition to the above-mentioned complications, microcatheter retention may occur between 3% and 8% of cases [98]. Onyx is a non-adhesive agent, so the initial expectation was a decrease in microcatheter retention with the use of this agent; however, clinical practice demonstrated the contrary. This was explained by the longer injection times and the need for a significant reflux to form a plug for efficient Onyx injection [61]. The incidence of microcatheter retention has decreased due to the introduction of new detachable tip microcatheters like Apollo (Medtronic, Irvine, CA, USA) and Sonic (Balt, Montmorency, France) [99]. 

### 3.4. Things to Consider

Timing of treatment: If the patient is symptomatic, most interventionalists will delay treatment until the patient recovers, unless other surgical indications are present, including large hematoma or ruptured intranidal aneurysm [4]. Hemorrhage may lead to a loss of vascular autoregulation in the surrounding region. Therefore, most surgeons wait a minimum of 4 weeks before treating to avoid postprocedural edema and bleeding [4]. In a study that evaluated the safety of delaying AVM treatment in clinically stable patients with a new hemorrhagic presentation, delaying intervention for at least 4 weeks after the initial hemorrhage subjected the patient to a low (<1%) risk of rehemorrhage [100]. 

Follow-up: Potts et al. and his colleagues did an extensive review of multiple series of AVMs cured with embolization [101]. There were a total of 668 patients with immediate angiographic cure with embolization. Of these, 4.5% had reported recurrence on follow-up angiography, highlighting the importance of angiographic follow-up after complete obliteration.

## 4. Arteriovenous Fistulas 

### 4.1. Background 

#### 4.1.1. Dural Arteriovenous Fistulae

Dural arteriovenous fistulae (dAVF) accounts for approximately 10–15% of all vascular malformations [102]. Unlike AVMs, which are suspected to be congenital, most dAVFs are considered to be acquired after trauma, surgery, venous stenosis, or sinus thrombosis [4,103]. They are most commonly located in the posterior fossa and cavernous sinus [4]. Like AVMs, they most commonly present with intraparenchymal hemorrhage; however, SAH or a combination of the two is also possible [4]. Borden et al. classified dAVFs into three groups based on the pattern of venous flow: type I dAVFs drain directly into venous sinus; type II dAVFs drain into venous sinuses but also have retrograde drainage into subarachnoid (cortical) veins; and type III dAVFs drain directly into subarachnoid veins [104]. On the other hand, Cognard et al. classified dAVF into five groups [105]. Drainage patterns that carry a lower risk for intracranial hemorrhage including antegrade outflow into a venous sinus (type I), with possible reflux into another sinus (type IIA). Higher risk lesions have outflow into a venous sinus with subsequent reflux into cortical veins (type IIB and IIC), direct outflow into cortical veins (type III), presence of venous varices (type IV), or drainage into perimedullary spinal veins (type V). DAVFs of the posterior fossa have an estimated risk of hemorrhage of 15% while those in the anterior fossa may have a risk from 65% to 80% [105]. 

#### 4.1.2. Brain Arteriovenous Fistulae

Brain arteriovenous fistulas (BAVFs) are rare lesions accounting for only 1.6–4.7% of all brain AV malformations [106,107]. They are characterized by an immediate arteriovenous transition without a capillary bed or “nidus” (vs. AVMs) [108]. This direct connection between an arterial feeder and draining vein creates conditions for rapid high flow causing its pathologic features. BAVFs can result from trauma, congenital or part of syndromes such as Klippel-Trenaunay-Weber or Rendu-Osler-Weber (hereditary hemorrhagic telangiectasia) [65,109,110,111,112,113]. 

BAVFs differ from dural arteriovenous fistulas (dAVFs) in that they derive their arterial supply from pial or cortical arterial vessels, and the lesion does not lie within the dural leaflets [109,110]. BAVFs can cause increased intracranial pressure, seizures, cerebral hemorrhage, neurologic deficit, cardiac failure in neonates and infants and intracranial bruit. However, BAVFs can also asymptomatic occasionally [114,115,116,117,118]. 

### 4.2. Management 

Not all fistulas require treatment [4]. Fistulas with antegrade, low flow into a dural sinus, even with reflux into an adjacent sinus, may have a benign course with low risk of hemorrhage. Some of those fistulas may thrombose spontaneously. However, even benign fistulas can have disabling symptoms, so treatment is sometimes warranted despite the low risk of hemorrhage [4]. 

Management options are typically surgery, endovascular therapy, or a combination of the two. The treatment of choice is highly dependent on the safe access to the fistula and ability to avoid sacrificing normal draining veins of the brain [4]. 

Endovascular therapy is a safe and effective way to treat lesions when surgical approach is risky, or when lesions are located at a deep or inaccessible location. Endovascular treatments include transarterial embolization and transvenous embolization with several different agents such as balloons, coils, glue (NBCA), and Onyx [109,119,120,121,122,123]. Coils are the favored agents to use via transvenous approach to a fistula. They are tightly packed by intermixing coils of varying sizes. On the arterial side, the high flow lesions can be slowed using the Berenstein Liquid Coils so a glue can be administered with diminished risk of pulmonary embolism [4]. If a transvenous route is not possible, glue may be the best option. They may be successful to occlude arterial pedicles proximal to and across the fistula [4]. Migration of the glue into the draining veins may block venous outflow resulting in immediate hemorrhage [124]. Onyx is a recent liquid embolic agent that can be used to slowly occlude small AV shunts in a more controlled way than the glue [124]. However, in some very high flow shunts, Onyx can migrate through the fistula into the distal draining veins causing immediate hemorrhage. Blocking the flow of the large high flow shunts using a microballoon to enable gradual occlusion using Onyx agent has been reported in the literature [119,122].

While endovascular techniques are often considered first-line therapy for the treatment of dAVFs, surgery remains an alternate effective and safe option. In few locations, such as anterior cranial fossa and ethmoidal dAVF, surgery is thought to be more successful than endovascular approaches. However, surgery is usually reserved for cases in which endovascular approaches have failed to completely cure the lesion. 

### 4.3. Complications 

The reported rate of complications in endovascular treatment of cerebral arteriovenous malformation, including BAVF, ranges between 3% and 25% [125,126]. According to published studies, the reported mortality rates associated with embolization are 2% or less [125]. 

Although rarely symptomatic, pulmonary emboli from the used embolic agents are fairly common [4]. 

## 5. Conclusions

This article highlights the endovascular treatment for the most common causes of intracerebral and subarachnoid hemorrhages. Endovascular therapy for intracranial aneurysms has an excellent safety profile and outcome, with similar or even better results compared with surgical clipping in selected patient populations. Flow diverting stents and intrasaccular flow disruptors offer relatively high occlusion rates for the treatment of wide necked aneurysms and overcome the shortcomings of coiling. Many endovascular embolizing agents such as NBCA and Onyx can be used in the treatment of arteriovenous malformations and fistulae with good outcomes.

## Figures and Tables

**Table 1:** Comparing treatment modalities of intracranial aneurysms.

	Clipping	Coiling	Stent-Coiling	Flow Diversion	Flow Disruption
Indications/ advantage	-Younger Patients (<50 years) -Unfavorable vascular anatomy - Higher occlusion rate	- Narrow Neck (<4 mm) - Lower complication rate compared to clipping	- Wide-Neck - Near or from bifurcation location - Used to improve occlusion rate after coiling, prevent coil herniation, and reduce retreatment rate	- Wide-Neck - Large (>12 mm)/Giant (>25 mm) aneurysms - Tandem aneurysms	-Wide-Neck -Neck >4 mm -Dome-to-neck >1 and <2 Dome size 3–10 mm - Saccular aneurysms - Bifurcation location - Dual antiplatelet medication is not necessary
Disadvantages	- Vasospasm - Stroke - Seizures - Bleeding	- Higher recanalization rate (especially bifurcation aneurysms and mainly basilar tip aneurysms). - Large, giant, fusiform, and large neck aneurysms are considered a challenge	- Thromboembolism - Procedure related permanent neurological deficit and mortality rate were higher in stent assisted coiling compared to coiling alone - Require antiplatelets	- Thromboembolic complications (needs dual anti-platelet therapy; limitation for ruptured aneurysms)	- Complications tend to occur more often in aneurysms with an unfavorable ratio between height and neck width - Cannot be placed in aneurysms with unfavorable neck angle

## Data Availability

Not applicable.
